# Handgrip strength as a surrogate marker of lean mass and risk of malnutrition in paediatric patients

**DOI:** 10.1016/j.clnu.2021.08.005

**Published:** 2021-09

**Authors:** Shona Mckirdy, Ben Nichols, Sarah Williamson, Konstantinos Gerasimidis

**Affiliations:** Human Nutrition, School of Medicine, Dentistry and Life Sciences, University of Glasgow, New Lister Building, Glasgow Royal Infirmary, G31 2ER, Glasgow, UK

**Keywords:** Handgrip strength, Children, Body composition, Paediatric yorkhill malnutrition score, Nutrition risk

## Abstract

**Background & aims:**

The use of handgrip strength (HGS) as a proxy of nutritional status in sick children has not been studied. This study created HGS centile charts in healthy children and explored the utility of HGS z-scores as markers of body composition and screening of malnutrition risk in sick children.

**Methods:**

Data from 535 healthy children aged 5–16 years were used for the development of HGS centiles adjusted either for age or height. In 595 sick children, relationships between HGS z-scores with body composition, malnutrition risk (Paediatric Yorkhill Malnutrition Score-PYMS), length of hospital stay (LOS) and biomarkers of disease severity were explored. The use of HGS z-score to identify sick children in need of further dietetic assessment was investigated.

**Results:**

Children scoring at high malnutrition risk with PYMS had lower HGS z-scores for age (by 0.51 SD, p < 0.001) and height (by 0.46 SD, p = 0.001) than those who scored low. A HGS z-score at cut-offs of ˗0.81 SD and ˗1.2 SD for age and height, respectively, was predictive of need for dietetic intervention in sick children with sensitivity of 79% and 70% and specificity of 56% and 69%, respectively. HGS z-scores were predictive of fat free mass (FFM) in sick and healthy (all p < 0.001) children, while fat mass was not. HGS z-scores were inversely related with plasma CRP (rho, age: ˗0.21; height: ˗0.23, both p = 0.001). HGS was not predictive of LOS.

**Conclusion:**

HGS is predictive of FFM, could compliment assessment of malnutrition risk, and may help identify children for further dietetic intervention on admission to hospital.

## 1 Introduction

Handgrip strength (HGS) is a quantitative measure of muscle function. It is non-invasive, inexpensive, and fast to obtain making it suitable to use at the bedside in routine clinical practice. In adults, HGS has been studied as an estimate of functional capacity [1], a predictor of frailty and risk of falls in elderly [[2], [3], [4], [5]], as a proxy of lean mass, and is a proposed component to the definition of malnutrition [1,[6], [7], [8]].

Likewise, the relationships between HGS with cardiovascular health, bone health, and total muscle mass have been studied in otherwise healthy children [[9], [10], [11]]. Previous research has also explored the use of HGS as a marker of muscle function in neurological and musculoskeletal disorders [[12], [13], [14], [15]] and its relationship with muscle mass in chronic kidney disease and cystic fibrosis [[16], [17], [18]]. Whether HGS could be used as a screening method of malnutrition risk in sick children admitted to hospital has not yet been studied.

The purpose of the study was to create centile charts for HGS for healthy children aged between 5 and 16 years old. These centile charts were then used to calculate HGS z-scores for sick children, and to explore associations with body composition, risk of malnutrition, length of hospital stay (LOS) and blood markers of disease severity in sick children. Lastly, we tested the performance of HGS in identifying hospitalized children in need of further dietetic assessment and intervention.

## 2 Subjects & methods

### 2.1 Healthy children for development of HGS centile charts

For development of the HGS centiles, eligible participants (5–16 years) were recruited from a range of schools and youth clubs in the areas of Greater Glasgow and Dumfries between the years 2005–2017. Participants with acute illness or chronic illness, the latter defined as requiring regular visits to health professionals or treatment, were not included.

### 2.2 Paediatric patients

The present study used existing data from sick children (5–16 years) who were recruited in previous studies on development and validation of paediatric malnutrition screening tools or other nutrition related research, and for which HGS measurements had been obtained [[19], [20], [21]]. Participants included surgical and medical inpatients from a large tertiary paediatric referral hospital in Glasgow [20,21] as well as children who were attending follow-up outpatient gastroenterology clinics in the same hospital [19] between the years 2008–2014. Patients in critical care and in high dependency unit were excluded in these previous studies, as well as children who, due to their condition, were deemed unable to provide reliable measurements of HGS (e.g. children with severe cerebral palsy). Patients were classified into specialties based on their primary reason of admission or background condition. Children from the inpatient wards were screened for risk of malnutrition using the Paediatric Yorkhill Malnutrition Score (PYMS) [22]. The Paediatric Yorkhill Malnutrition Score was developed and validated to identify children at risk of malnutrition on hospital admission and refer them to the hospital dietitians for further nutritional assessment. PYMS assesses BMI, parental reports of unintended weight loss, changes to nutritional intake for more than 7 days, and the predicted effect of admission condition on nutritional status parameters [21]. Participants with a PYMS score of zero were classed as low risk, those with a score of one were at medium risk and those with a score of two or more were at high risk of malnutrition. A proportion of the same patients were also assessed by a clinical research dietitian using standard assessment methodology applied in routine clinical practice including growth centiles, dietary history, and clinical review. The outcome of dietetic assessment was classified as a binary response of patients who needed further review and dietetic intervention or not.

### 2.3 Anthropometry and HGS measurement

Standing height was measured to the nearest millimetre without shoes using a portable stadiometer (Seca model 213). Weight and body composition measurements were taken using Tanita scales (TBF-300) with children wearing light clothing and accounting 0.5 kg for residual clothing weight. For anthropometric measurements, z-scores were calculated using the LMS Growth and the WHO-UK growth references [23]. Participants with a height z-score of < -2 SD were classed as having a short stature. Those with a BMI z-score of < -2 SD were classed as underweight, while those with a z-score of >2 SD were classed as obese. Using the raw impedance measurements (Ohms), indices of fat mass (FM) and fat free mass (FFM) standardised for gender, age and height and accounting for the biological variation on FFM hydration with age were calculated for children 5–13 years of age [24].

### 2.4 Handgrip measurements

HGS measurements were taken using a Takei Analogue (5001) Hand Grip Dynamometer. Measurements were taken three times from each hand with the participant seated and resting the dynamometer on their lap and three times from each hand while standing with the scale by their side. Thirty seconds of rest were allowed between measurements on the same hand to reduce the risk of muscle fatigue affecting the measurements. Children were asked which hand they write with and this was recorded as their dominant side.

Maximum grip strength from the dominant hand while standing and while sitting was calculated and the same was calculated for the non-dominant hand. The maximum HGS while standing and while sitting was then calculated, regardless of which hand this measurement was from. In sick children, the same procedures were applied, but due to their clinical condition (e.g. unable to stand or plaster or intravenous cannulas were in situ) some patients could not perform all measurements. Like the healthy children, the maximum HGS was then calculated for either of the groups.

### 2.5 Statistical analysis

Reference centiles for HGS for age and height and for each gender separately were plotted using the Generalized Additive Models for Location, Scale and Shape package (GAMLSS) [25] in R version 4.0.2. Models were generated using the different combinations of input variables to the GAMLSS function, pertaining to model distribution, degrees of freedom, and smoothing method. The final model selected for each dataset was chosen using the optimal Akaike information criterion and root mean square error values. In instances where these values differed by less than 5% between models, the simpler model was selected. Upon these centiles, z-scores of HGS were calculated for the sick children. Univariate linear regression was performed to determine whether body composition was predictive of HGS z-scores. Paired t-tests were carried out to determine if there was a significant difference in maximum HGS between the dominant and non-dominant hand, and if HGS was affected by body position (i.e. standing vs sitting). Differences between malnutrition risk categories, according to PYMS, and between disease specialties (with >10 patients enrolled per specialty) and against healthy controls were estimated using analysis of variance. A receiver operating characteristic (ROC) curve was plotted to identify HGS z-score cut-off values under which patients should be referred for further dietetic review and intervention. Sensitivity, specificity, positive and negative predictive values at these cut-offs were estimated for the entire cohort of patients and for specialties with more than 10 patients enrolled and with more than four patients assessed by the dietitians as in need for further review and intervention. The ability of low HGS z-score (i.e. < ˗2 SD) to predict LOS was explored using survival analysis and Kaplan–Meier curves. Statistical analysis was performed with MINITAB 19.1.1, Coventry UK and MedCalc 19.7, Ostend, Belgium.

### 2.6 Ethical considerations

All participants and their legal guardians were informed about the study and signed informed consent. Children unable to provide informed consent or assent (e.g. children with learning difficulties) were excluded according to the Good Clinical Practice standards for research. For the healthy children, the study was approved by the Research Ethical Committee of the Medical School of the University of Glasgow (Project No: 200130025) and for the sick children by West of Scotland Research Ethical Committee of the National Health Service.

## 3 Results

### 3.1 Descriptive characteristics

**Fig. 1:**
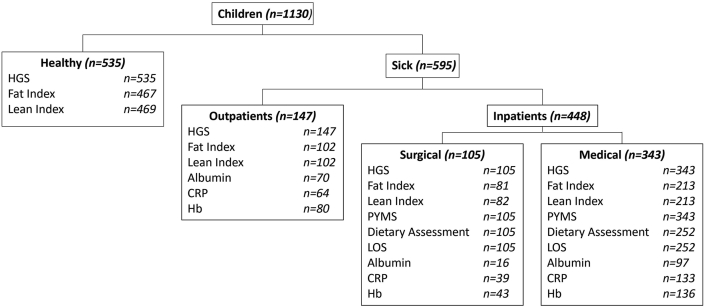
Flowchart of data collection in sick and healthy children. HGS = handgrip strength, CRP = C-reactive protein, Hb = haemoglobin, PYMS = paediatric Yorkhill malnutrition score, LOS = length of hospital stay.

535 healthy (mean, SD age: 10.0, 2.7 y; boys: n = 316, 59%) and 595 sick (mean, SD age: 10.4, 3.0 y; boys: 325, 55%) children were included in the study. 343 (58%), 105 (17%) and 147 (25%) of the sick children were from the medical inpatient, surgical inpatient, and gastroenterology outpatient wards, respectively (Table 1, Fig. 1). Information on the disease specialties of patients is presented in Supplementary Table 1. Eleven (2.1%) of the healthy children and 32 (5.4%) of the sick children had a short stature. Eight (1.5%) of the healthy and 29 (4.9%) of the sick children were underweight (BMI z-score < ˗2 SD) and 46 (8.6%) and 58 (9.7%) respectively were obese (BMI z-score > 2SD).

**Table 1:** Characteristics of healthy children used for the development of handgrip strength centiles and of paediatric patients of a tertiary paediatric hospital.

Variable	Healthy		Sick		p
	N	Mean (SD)	N	Mean (SD)	
Height (cm)	535	139 (16.4)	595	140 (17.7)	0.459
Weight (kg)	535	36.5 (14)	594	37.2 (15.2)	0.405
Age (years)	536	9.96 (2.75)	595	10.4 (3)	0.006
Height z-score (SD)	535	0.18 (1.11)	595	−0.11 (0.05)	<0.001
Weight z-score (SD)	535	0.37 (1.15)	594	0.08 (1.32)	<0.001
BMI (kg/m^2^)	535	18.2 (3.49)	594	18.2 (3.95)	0.757
BMI z-score (SD)	535	0.37 (1.15)	594	0.16 (1.34)	0.005
LOS (days)	–	–	357	5.08 (10.9)	–
Albumin (g/L)	–	–	183	37.2 (5.13)	–
CRP (mg/L)	–	–	236	26.8 (50.9)	–
Hb (g/dL)	–	–	259	12.7 (1.57)	–

### 3.2 Handgrip strength in healthy children and development of centile charts

In healthy children, the median coefficient of variation (%) of the three HGS measurements varied between 5.4% and 5.9% for either of the hand and trial position. Measurements from the dominant hand and sitting posture were higher than those from the non-dominant hand and standing posture, respectively (Supplementary Fig. 1).

The maximum value of all measurements and trial positions was subsequently used to plot HGS centile charts (Supplementary Figs. 2–5). As expected, age and height were predictive of HGS. Using the Box–Cox Cole and Green distribution function in GAMLSS package in R, two HGS centile charts were developed: one for height and another for age, and for each gender separately. The cut-off values for the major centiles are presented in Supplementary Table 2.

### 3.3 Differences in handgrip strength between healthy and sick children

**Fig. 2:**
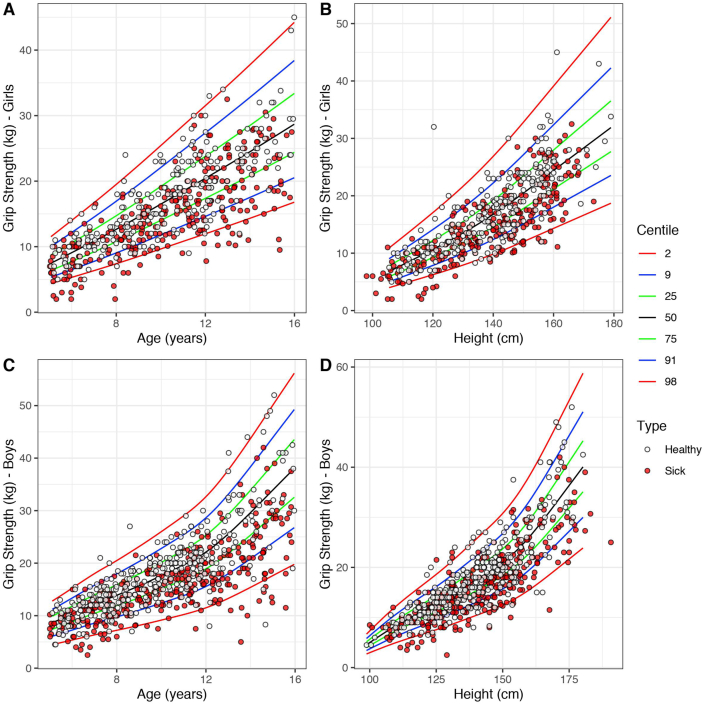
Handgrip strength (kg) centile charts adjusted for age (years) and height (cm) in girls (A & B) and boys (C & D).

Using the HGS centile charts from the healthy children, z-scores for age and height were computed for the group of sick children (Fig. 2). The R code and the data from the heathy children for the computation of HGS z-scores by independent researchers and health care professionals using their own data can be accessed here: https://doi.org/10.5525/gla.researchdata.1131.

HGS z-scores, either for age or height, were significantly lower in sick than in healthy children. On average, sick children had a HGS z-score for age of 0.75 SD (95% CI 0.64, 0.87, p < 0.001) and for height of 0.63 SD (95% CI 0.51, 0.75, p < 0.001) lower than healthy children (Fig. 2). Among the sick groups of children, HGS z-score for height was significantly lower in medical (p = 0.002) and surgical inpatients (p = 0.012) than the medical gastroenterology outpatients (Table 2). There was no difference between medical and surgical inpatients in mean HGS z-scores for height (p = 0.954). When the analysis was repeated using the age adjusted HGS z-score, no differences were observed between these groups. When assessing differences in HGS z-scores between specialties, HGS z-scores were significantly higher in gastroenterology patients compared to general medical and surgical specialties, and all significantly lower than healthy controls (Supplementary Table 1). However, since the majority of the gastroenterology patients were outpatients, and all general medical and surgical patients were inpatients, this is likely a reflection of an inpatient compared with outpatient effect than an effect of disease specialty.

**Table 2:** Handgrip strength (HGS) z-score adjusted for age (years) and height (cm) according to inpatient/outpatient group, malnutrition risk assigned by PYMS and dietetic assessment outcome and BMI class for sick children.

		N	HGS_z_age	HGS_z_height
Group	*Surgical inpatient*	105	−0.91 (1.10)	−0.74 (1.15) ∗
	*Medical inpatient*	343	−0.75 (1.06)	−0.71 (1.04) †
	*Medical outpatient*	147	−0.64 (0.96)	−0.36 (1.03) ∗ †
PYMS Malnutrition Risk	*Low*	286	−0.65 (1.08) ∗	−0.73 (1.23) ∗
	*Medium*	73	−0.96 (1.27)	−0.93 (1.42)
	*High*	89	−1.16 (0.97) ∗	−1.16 (1.11) ∗
Dietetic assessment outcome	*Low*	324	−0.74 (1.03) ∗	−0.66 (1.05) ∗
	*High*	33	−1.61 (1.22) ∗	−1.50 (1.08) ∗
BMI Class	*Underweight*	29	−1.72 (0.66) ∗ †	−1.31 (1.05) ∗ †
	*Normal*	498	−0.74 (1.03) ∗ ‡	−0.63 (1.05) ∗ ‡
	*Obese*	58	−0.34 (1.02) † ‡	−0.28 (1.14) † ‡

Data are presented as mean (SD). Within each HGS z-score column, values that share a symbol are significantly different (p < 0.05).

Measurements of plasma C-reactive protein (CRP) were available for 236 sick children. An inverse correlation was observed between plasma CRP concentration and HGS z-score (rho, age: ˗0.21; height: ˗0.23, both p = 0.001) suggesting ongoing systemic inflammatory response was associated with a lower HGS z-score. In contrast, haemoglobin and serum albumin levels were not related with HGS z-score. Neither HGS z-score for age nor for height were correlated with or predictive of LOS (all p > 0.05). Using survival analysis, a HGS below the 2nd centile was not predictive of LOS, either for age or height adjusted z-scores (p = 0.44 and p = 0.612, respectively) (Supplementary Fig. 6).

### 3.4 Handgrip strength as a surrogate marker of body composition in healthy and sick children

BMI z-score was positively correlated with HGS z-score for age and for height in both the sick (rho, age: 0.18, height: 0.18, both p < 0.001) and healthy (rho, age: 0.22, height: 0.17, both p < 0.001) children. When body composition estimates were used instead of anthropometry, these correlations improved for FFM z-scores (rho, sick children age: 0.24, height: 0.33; healthy children age: 0.29, height: 0.31; all p < 0.001) but were non-significant for FM z-scores (all p > 0.05). Underweight children had lower HGS z-scores for age and height than normal weight children (Table 2). This was independent of the health status of the children. Mean HGS of obese children was not significantly different compared to normal weight children for either age or height amongst the healthy children (all p > 0.05). In sick children, age adjusted HGS z-scores were higher in obese children than normal weight children by 0.4 SD (p = 0.02) (Table 2).

HGS z-scores of either age or height predicted 4.28% and 1.84% of variance of BMI z-scores in healthy children and 3.47% and 3.64% in sick children, respectively. These predictions became greater for FFM z-scores, with age and height adjusted HGS predicting 9.54% and 10.4% of variance in healthy children and 5.4% and 10.4% of variance in sick children, respectively. Only 0.91% of FM variance was predicted by height adjusted HGS in sick children (p = 0.03). From the 21 healthy children and 62 sick children with a low (i.e. <-2 SD) FFM z-score, less than 10% and 15% had also a low (i.e. <-2SD) HGS z-score. ROC analysis and use of different thresholds for HGS z-score improved sensitivity to screen children with low FFM. However, in sick and healthy children, the false positive rate was high at 73% and 93% for age and height adjusted HGS measurements, respectively (data not presented).

### 3.5 Handgrip strength, dietetic assessment outcome and malnutrition risk

Using PYMS, 448 sick children had been screened for malnutrition risk. Of them, 89 (20%) scored at high, 73 (16%) at medium and 286 (64%) at low malnutrition risk. Children screened at high PYMS malnutrition risk had lower HGS z-scores by 0.51 SD (p < 0.0001) and 0.46 SD p = 0.001) for age and height, respectively, compared to children at low risk (Table 2).

Of the children above, dietary assessment was available for 357. Thirty-three (9.2%) were assessed to require further dietetic review and intervention. In these children, mean HGS z-scores for age and height were low (˗1.6 [1.2] and ˗1.5 [1.1], respectively) and on average significantly lower (p < 0.0001) by 0.8 SD than children who were not deemed to require dietary intervention (Table 2).

**Fig. 3:**
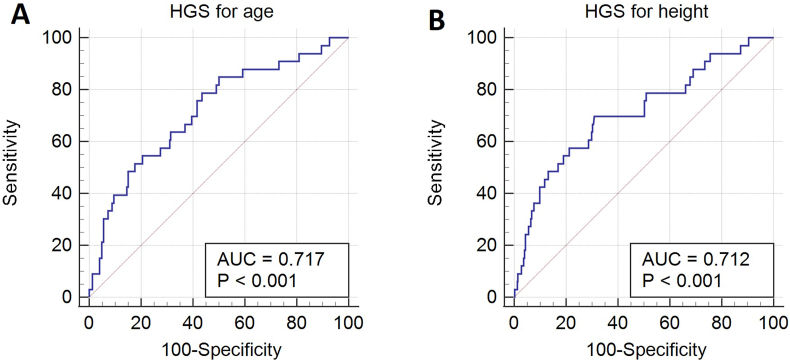
Receiver operating characteristic (ROC) curve for handgrip strength (HGS) adjusted for age (A) and height (B) as a tool to detect high malnutrition risk in sick children.

Using ROC analysis, the areas under the curve of HGS z-scores for age and height were 0.72 (SEM, 0.05) and 0.71 (SEM, 0.05), both p < 0.001 (Fig. 3). For the age adjusted HGS z-scores, a criterion value at −0.81 would give HGS sensitivity, specificity, positive predictive value (PPV) and negative predictive value (NPV) of 79%, 56%, 16%, and 96%, respectively, to screen children who need dietary intervention. Likewise, for the height adjusted HGS z-scores, a criterion value at −1.2 would give HGS sensitivity, specificity, PPV and NPV of 70%, 69%, 19% and 96%, respectively. Subset ROC analysis according to patients’ specialty showed that the performance of the HGS was particularly good for patients from the gastroenterology wards (HGS for age: criterion value = −1.22, sensitivity = 69%, specificity = 88%, PPV = 75%, NPV = 84%; HGS for height: −1.21, 69%, 83%, 69%, 83%, respectively) than for patients from general medical (HGS for age: −0.67, 91%, 50%, 15%, 98%; HGS for height: −1.77, 64%, 88%, 35%, 96%, respectively) and surgical (HGS for age: ˗2, 80%, 85%, 25%, 99%; HGS for height: −1.46, 80%, 74%, 16%, 98%, respectively) wards (Supplementary Fig. 7).

## 4 Discussion

The current study aimed to develop centile charts of HGS in healthy children (Fig. 2) and subsequently evaluate their use as nutritional status indices in a large cohort of sick children. HGS is a measurement which can be performed quickly and non-invasively with paediatric patients, so its relationship with body composition compartments and its usefulness as a screening tool for dietary intervention make it attractive for use in routine clinical practice. In the current study, BMI z-score was positively correlated with HGS z-score for age and height in both sick and healthy children, consistent with findings from other studies [26,27]. However, when considering body composition, a greater variance of FFM z-scores was explained by HGS z-scores than by BMI, while only the HGS z-scores adjusted for age were slightly predictive of FM z-scores in sick children. This suggests that HGS can offer important insights into the body composition features and specifically muscle mass of a healthy or sick child. As muscle strength is known to be lost when malnutrition and chronic inflammation occur and FM is not a major confounding factor in HGS measurement, the current data suggest that HGS might be a useful tool in the assessment of muscle stores over time and changes post-intervention, with little interference from changes in fat levels, particularly when bedside body composition is not available. Nevertheless, only a small proportion of children with low FFM also presented low measurements of HGS and vice versa, thus suggesting that other parameters explain a low HGS in a child, including disease severity.

Children with chronic illness often experience a greater loss of muscle mass due to undernutrition, chronic inflammation and the side-effects of certain medications [28]. Therefore, malnutrition screening tools, such as PYMS, may overlook a disproportionate muscle to fat mass when only body mass and BMI are considered. Thus, a tool which can infer the FFM of a paediatric patient could be a useful addition into the process of malnutrition screening and further inform any necessary dietary interventions. In the current study, the sick children who required dietary intervention had significantly lower HGS z-scores for both age and height compared to the sick children who did not. This finding indicates that patients with low HGS z-scores for age and height may be at higher risk of malnutrition and thus more likely to require dietary intervention. Analysis using ROC found that measuring HGS and adjusting for age or height might be a practical tool for identifying a need for further dietetic assessment and intervention in paediatric patients. Sick children with a HGS z-score for age or height of −0.81 and −1.2 respectively, could be referred for dietary intervention with good sensitivity and moderate specificity (Fig. 3). However, the positive predictive validity of HGS z-scores was weak, meaning that the proportion of false positive screens would be significant if HGS were to be used in isolation. Thus, to screen for malnutrition risk on admission to hospital, HGS may be better used alongside existing screening tools, such as PYMS [29]. However, the combined use of these tools to assess malnutrition and body composition was not investigated in this study, so assessing whether PYMS would benefit with the addition of HGS measurement is an area for future research.

It is important to note that plasma CRP was negatively correlated with HGS z-scores for age and height, and inpatients, who are more likely to be acutely unwell, had lower HGS than outpatients from a gastroenterology ward. Hence, disease severity, particularly in conditions associated with ongoing inflammatory response, must also be considered as these factors may influence HGS measurement predictive validity. Unfortunately, further information on disease severity was not collected and whether the patients suffered from acute or chronic disease was difficult to ascertain. However, the findings with CRP indicate that HGS measurements might not be a reliable indicator of FFM or malnutrition in patients with severe or active disease. Ideally, measurements in sick children should not be performed in the active phase of the disease, but rather in recovery. In turn, this is likely to reduce the false positive rate of children in need of dietary intervention.

Another objective of this study was to develop reference centiles of HGS for children of British background (Supplementary Figs. 1–4). Reference ranges for HGS in children have previously been developed in the literature and our findings are well in agreement where HGS increases with age and height [22,23,30,31]. In both boys and girls, a greater variation in grip strength was identified between children as age increased, likely due to genetic influence on physical development during puberty. This was also seen in the height charts as children grew taller and highlights the importance of adjusting HGS for gender and height, with the latter also partially correcting for any effect delayed puberty may have on HGS measurements [32].

Several testing conditions can influence the measured HGS force, including the position and angle of the arm and elbow, the number of trials, and the allocated rest period between trials [33]. In accordance with previous research, this study found that HGS measurements from the dominant hand were significantly higher than those from the non-dominant hand (Supplementary Fig. 5). This is a common finding in literature, such that the average difference in strength between the dominant and non-dominant hand is commonly known as the “10% rule” [34]. In adults, it has been found that this “10% rule” exists only in right-handed people, with HGS in left-handed people being equivalent between hands [35]. However, the effect of hand dominance on strength is less studied in children and in the current study, we showed that test position and handedness can introduce a measurement variation of approximately 5–6% on average.

A strength of this paper is that a large number of healthy children were recruited from both urban and rural areas, giving a large degree of diversity in the characteristics of children, thus making the reference data more representative of the general local healthy paediatric population. Sick children were all recruited from a single large tertiary children's hospital; however, they were recruited from a range of departments to diversify the disease profiles of children in the study.

The present study would have benefitted from additional patient information which could have been investigated for confounding the observed relationship between HGS and FFM and risk of malnutrition. This includes the chronicity of the condition, types of medication patients were receiving, pubertal staging and bone age, all of which have the potential to influence maximal HGS.

In conclusion, this study has produced HGS centile charts for males and females, corrected for both age and height. It was shown that HGS is predictive of FFM and might be used as a complimentary method to screen for nutritional risk and the need for further review and dietetic intervention on admission to hospital. However, disease severity, particularly systemic inflammatory response, may confound the relationship between FFM and HGS in sick children and increase false screening of children at risk of malnutrition; hence, the timing of measurement in this group of children is important.

## Funding statement

This research did not receive any specific grant from funding agencies in the public, commercial, or not-for-profit sectors. Dr Ben Nichols was part-funded by the Biotechnology and Biological Sciences Research Council (Ref: BB/R006539/1).

## Author contribution

Shona Mckirdy: Performed statistical analysis and drafted the manuscript; Sarah Wiliamson: Contributed to drafting the manuscript and collected part of the data; Ben Nichols: Created the centile charts and calculated z-scores for participants; Konstantinos Gerasimidis: Conceived the study, gained ethical permission, collected part of the data, revised the draft manuscript and supervised all research activities.

## Conflicts of interest

KG reports personal fees from Nutricia, research grants and personal fees from Nestle, personal fees from Dr Falk, Abbott, and Baxter. The other authors have no conflict of interest to declare.
