# Supplementary material for: Handgrip strength as a surrogate marker of lean mass and risk of malnutrition in paediatric patients
Source: Clin Nutr. 2021 Sep;40(9):5189–95. doi: 10.1016/j.clnu.2021.08.005 (PMC8460712; doi:10.1016/j.clnu.2021.08.005)
Supplement: Multimedia component 1 [file mmc1.docx]

Supplementary Material

**Supplementary Table 1**: Grip strength z-score adjusted for age (years) and height (cm) according to disease specialty in the sick children. Data are presented as mean (SD). Between group comparisons and comparisons to healthy children were performed with specialties of more than 10 patients.

| **Specialty** | **N** | **GS_z_age** | **GS_z_height** |
| --- | --- | --- | --- |
| Burns | 4 | 0.08 (1.34) | 0.12 (1.34) |
| Cardiology | 3 | -1.03 (0.48) | -1.09 (0.34) |
| Dermatology | 8 | -0.59 (0.79) | -0.55 (0.99) |
| Endocrinology | 12 | -0.74 (0.91)* | -0.69 (1.04)* |
| Ear/Nose/Throat | 6 | -0.28 (0.65) | -0.43 (0.58) |
| Gastroenterology | 195 | -0.71 (1.01)* | -0.47 (1.09)* ^ab^ |
| General Medical | 139 | -0.75 (0.08)* | -0.85 (0.95)* ^b^ |
| Haematology | 1 | -2.14 | -1.49 |
| Neurology/Neurosurgery | 30 | -0.56 (1.24)* | -0.58 (1.17)* |
| Oncology | 13 | -0.89 (1.13)* | -0.97 (1.11)* |
| Ophthalmology | 1 | 0.38 | 1.01 |
| Orthopaedics | 4 | -0.51 (0.63) | -0.17 (1.47) |
| Plastics | 1 | -1.14 | 0.08 |
| Renal | 11 | -0.71 (1.16)* | -0.6 (0.98)¥ |
| Respiratory | 69 | -0.72 (0.98)* | -0.42 (1.01)* |
| Rheumatology | 3 | -0.71 (0.78) | 0.14 (0.84) |
| Surgical | 86 | -1.05 (1.21)* | -0.89 (1.19)* ^a^ |
| Urology | 3 | -0.67 (0.51) | -0.94 (0.49) |

*For between group comparisons, values which share a letter are significantly different (p<0.05).*

*Symbols indicate differences between patient group and healthy children; *p<0.05, ¥ p=0.056*

**Supplementary Table 2:** Major cut-off values for handgrip strength centile charts.

1. Handgrip strength for height for girls

| **Height** | **2^nd^** | **9^th^** | **25^th^** | **50^th^** | **75^th^** | **91^st^** | **98^th^** |
| --- | --- | --- | --- | --- | --- | --- | --- |
| **105** | 3.920604 | 4.935988 | 5.804673 | 6.68328 | 7.658716 | 8.861251 | 10.71023 |
| **106** | 4.073321 | 5.128257 | 6.030779 | 6.943611 | 7.957042 | 9.206419 | 11.12742 |
| **107** | 4.22609 | 5.320591 | 6.256962 | 7.20403 | 8.255469 | 9.551703 | 11.54475 |
| **108** | 4.378968 | 5.513061 | 6.483305 | 7.464632 | 8.554107 | 9.897232 | 11.96238 |
| **109** | 4.531998 | 5.705724 | 6.709876 | 7.725497 | 8.853045 | 10.24311 | 12.38042 |
| **110** | 4.68526 | 5.898679 | 6.936788 | 7.986755 | 9.152434 | 10.58951 | 12.7991 |
| **111** | 4.838862 | 6.092062 | 7.164205 | 8.248594 | 9.45249 | 10.93667 | 13.21871 |
| **112** | 4.992917 | 6.286016 | 7.392293 | 8.511206 | 9.753429 | 11.28487 | 13.63955 |
| **113** | 5.147532 | 6.480673 | 7.621208 | 8.77477 | 10.05546 | 11.63432 | 14.06193 |
| **114** | 5.30277 | 6.676116 | 7.851046 | 9.039397 | 10.35871 | 11.98519 | 14.486 |
| **115** | 5.458672 | 6.872394 | 8.081867 | 9.305156 | 10.66326 | 12.33755 | 14.91189 |
| **116** | 5.615278 | 7.06956 | 8.313733 | 9.572117 | 10.96918 | 12.69151 | 15.33971 |
| **117** | 5.772635 | 7.267669 | 8.546707 | 9.840355 | 11.27657 | 13.04716 | 15.76957 |
| **118** | 5.930801 | 7.466798 | 8.780881 | 10.10997 | 11.58554 | 13.40465 | 16.20165 |
| **119** | 6.089841 | 7.667028 | 9.016349 | 10.38108 | 11.89622 | 13.76411 | 16.63611 |
| **120** | 6.24982 | 7.868439 | 9.253206 | 10.65379 | 12.20873 | 14.12568 | 17.07313 |
| **121** | 6.410817 | 8.071132 | 9.491571 | 10.92824 | 12.52323 | 14.48957 | 17.51294 |
| **122** | 6.572943 | 8.275246 | 9.731608 | 11.2046 | 12.83993 | 14.856 | 17.95584 |
| **123** | 6.736313 | 8.480927 | 9.973486 | 11.48309 | 13.15907 | 15.22524 | 18.40213 |
| **124** | 6.901042 | 8.688319 | 10.21738 | 11.7639 | 13.48086 | 15.59756 | 18.85213 |
| **125** | 7.067271 | 8.897599 | 10.46349 | 12.04726 | 13.80558 | 15.97327 | 19.30623 |
| **126** | 7.235164 | 9.108974 | 10.71206 | 12.33346 | 14.13355 | 16.35274 | 19.76488 |
| **127** | 7.404888 | 9.322654 | 10.96335 | 12.62278 | 14.4651 | 16.73634 | 20.22853 |
| **128** | 7.576609 | 9.538848 | 11.21759 | 12.91551 | 14.80055 | 17.12446 | 20.69763 |
| **129** | 7.750498 | 9.757772 | 11.47504 | 13.21193 | 15.14023 | 17.51748 | 21.17266 |
| **130** | 7.926728 | 9.979644 | 11.73596 | 13.51234 | 15.48449 | 17.91579 | 21.65408 |
| **131** | 8.105472 | 10.20468 | 12.0006 | 13.81704 | 15.83366 | 18.31978 | 22.14237 |
| **132** | 8.286883 | 10.43307 | 12.26919 | 14.12628 | 16.18804 | 18.7298 | 22.63795 |
| **133** | 8.471029 | 10.66491 | 12.54183 | 14.44019 | 16.54776 | 19.14601 | 23.14099 |
| **134** | 8.657957 | 10.90025 | 12.81859 | 14.75884 | 16.91291 | 19.5685 | 23.65164 |
| **135** | 8.847716 | 11.13916 | 13.09954 | 15.08231 | 17.2836 | 19.99738 | 24.17002 |
| **136** | 9.040323 | 11.38165 | 13.3847 | 15.41064 | 17.65985 | 20.43271 | 24.69618 |
| **137** | 9.235744 | 11.62768 | 13.67403 | 15.74376 | 18.04159 | 20.8744 | 25.23002 |
| **138** | 9.433934 | 11.8772 | 13.96747 | 16.08161 | 18.42875 | 21.32234 | 25.77144 |
| **139** | 9.634852 | 12.13015 | 14.26494 | 16.42411 | 18.82123 | 21.77645 | 26.3203 |
| **140** | 9.838469 | 12.3865 | 14.5664 | 16.7712 | 19.21899 | 22.23666 | 26.87654 |
| **141** | 10.04477 | 12.64623 | 14.87184 | 17.12287 | 19.62198 | 22.70293 | 27.4401 |
| **142** | 10.25374 | 12.90931 | 15.18123 | 17.47909 | 20.03019 | 23.17523 | 28.01095 |
| **143** | 10.46535 | 13.17573 | 15.49453 | 17.83982 | 20.44357 | 23.65352 | 28.58903 |
| **144** | 10.67954 | 13.4454 | 15.81166 | 18.20494 | 20.86198 | 24.13763 | 29.17417 |
| **145** | 10.89623 | 13.7182 | 16.13247 | 18.57432 | 21.28527 | 24.62738 | 29.7661 |
| **146** | 11.11532 | 13.99403 | 16.45684 | 18.94779 | 21.71325 | 25.12256 | 30.3646 |
| **147** | 11.33669 | 14.27274 | 16.7846 | 19.32515 | 22.14569 | 25.6229 | 30.96935 |
| **148** | 11.56013 | 14.55405 | 17.11542 | 19.70605 | 22.58218 | 26.12792 | 31.57975 |
| **149** | 11.78541 | 14.83767 | 17.44895 | 20.09007 | 23.02224 | 26.63708 | 32.19515 |
| **150** | 12.01227 | 15.12329 | 17.78484 | 20.47679 | 23.46541 | 27.14984 | 32.8149 |
| **151** | 12.24049 | 15.41061 | 18.12273 | 20.86582 | 23.91122 | 27.66564 | 33.43833 |
| **152** | 12.46981 | 15.69932 | 18.46224 | 21.25673 | 24.35918 | 28.18394 | 34.06477 |
| **153** | 12.69999 | 15.98911 | 18.80304 | 21.6491 | 24.80883 | 28.70419 | 34.69357 |
| **154** | 12.93078 | 16.27968 | 19.14475 | 22.04254 | 25.25968 | 29.22583 | 35.32406 |
| **155** | 13.162 | 16.57078 | 19.48707 | 22.43668 | 25.71135 | 29.74842 | 35.95569 |
| **156** | 13.39347 | 16.8622 | 19.82979 | 22.83126 | 26.16352 | 30.27159 | 36.58803 |
| **157** | 13.62505 | 17.15376 | 20.17265 | 23.22603 | 26.6159 | 30.795 | 37.22065 |
| **158** | 13.85659 | 17.44526 | 20.51545 | 23.62071 | 27.06819 | 31.31831 | 37.85316 |
| **159** | 14.08801 | 17.73661 | 20.85808 | 24.0152 | 27.52026 | 31.84136 | 38.48534 |
| **160** | 14.31928 | 18.02778 | 21.2005 | 24.40945 | 27.97205 | 32.36408 | 39.11714 |
| **161** | 14.55039 | 18.31874 | 21.54266 | 24.8034 | 28.42349 | 32.88641 | 39.74846 |
| **162** | 14.78129 | 18.60945 | 21.88453 | 25.19702 | 28.87456 | 33.40831 | 40.37925 |
| **163** | 15.01203 | 18.89995 | 22.22615 | 25.59035 | 29.3253 | 33.92982 | 41.00958 |
| **164** | 15.24264 | 19.19028 | 22.56758 | 25.98346 | 29.77578 | 34.45103 | 41.63955 |
| **165** | 15.47315 | 19.48049 | 22.90886 | 26.37639 | 30.22607 | 34.97202 | 42.26925 |
| **166** | 15.70359 | 19.77061 | 23.25005 | 26.76922 | 30.67623 | 35.49287 | 42.89877 |
| **167** | 15.93401 | 20.0607 | 23.59118 | 27.162 | 31.12633 | 36.01364 | 43.52821 |
| **168** | 16.16442 | 20.35079 | 23.93233 | 27.55477 | 31.57644 | 36.53442 | 44.15765 |
| **169** | 16.39487 | 20.64092 | 24.27352 | 27.94761 | 32.0266 | 37.05527 | 44.78718 |
| **170** | 16.62536 | 20.93111 | 24.61478 | 28.34052 | 32.47687 | 37.57623 | 45.41684 |
| **171** | 16.85591 | 21.22137 | 24.95612 | 28.73353 | 32.92723 | 38.09731 | 46.04665 |
| **172** | 17.08652 | 21.51169 | 25.29754 | 29.12663 | 33.37771 | 38.61851 | 46.67661 |
| **173** | 17.31717 | 21.80209 | 25.63904 | 29.51982 | 33.82828 | 39.13984 | 47.30671 |
| **174** | 17.54787 | 22.09254 | 25.98061 | 29.91309 | 34.27895 | 39.66126 | 47.93694 |
| **175** | 17.77862 | 22.38304 | 26.32223 | 30.30642 | 34.72969 | 40.18278 | 48.56728 |
| **176** | 18.00938 | 22.67357 | 26.6639 | 30.6998 | 35.18048 | 40.70435 | 49.19768 |
| **177** | 18.24017 | 22.96413 | 27.00559 | 31.09321 | 35.63131 | 41.22597 | 49.82814 |
| **178** | 18.47097 | 23.2547 | 27.3473 | 31.48664 | 36.08217 | 41.74762 | 50.45863 |
| **179** | 18.70177 | 23.54528 | 27.68902 | 31.88008 | 36.53303 | 42.26928 | 51.08914 |

1. Handgrip strength for height for boys

| **Height** | **2^nd^** | **9^th^** | **25^th^** | **50^th^** | **75^th^** | **91^st^** | **98^th^** |
| --- | --- | --- | --- | --- | --- | --- | --- |
| **98** | 2.5449 | 3.195694 | 3.740113 | 4.271158 | 4.824521 | 5.442824 | 6.263223 |
| **99** | 2.760114 | 3.465944 | 4.056403 | 4.632356 | 5.232515 | 5.903106 | 6.792883 |
| **100** | 2.975119 | 3.73593 | 4.372384 | 4.993202 | 5.640112 | 6.362939 | 7.322028 |
| **101** | 3.189252 | 4.004823 | 4.687085 | 5.352587 | 6.046057 | 6.82091 | 7.849029 |
| **102** | 3.402016 | 4.271996 | 4.999775 | 5.709674 | 6.449407 | 7.275953 | 8.372661 |
| **103** | 3.613064 | 4.537015 | 5.309941 | 6.06388 | 6.849504 | 7.727325 | 8.892068 |
| **104** | 3.82183 | 4.799166 | 5.616753 | 6.414255 | 7.245273 | 8.173815 | 9.405858 |
| **105** | 4.027704 | 5.057688 | 5.919317 | 6.759778 | 7.635561 | 8.614123 | 9.912533 |
| **106** | 4.230079 | 5.311815 | 6.216737 | 7.099428 | 8.019216 | 9.046946 | 10.4106 |
| **107** | 4.428457 | 5.560923 | 6.508283 | 7.43237 | 8.395293 | 9.47122 | 10.89882 |
| **108** | 4.622782 | 5.804942 | 6.793873 | 7.758509 | 8.763686 | 9.886826 | 11.37707 |
| **109** | 4.813161 | 6.044006 | 7.073665 | 8.078028 | 9.1246 | 10.29399 | 11.84561 |
| **110** | 4.999797 | 6.27837 | 7.347954 | 8.391262 | 9.478417 | 10.69316 | 12.30494 |
| **111** | 5.182867 | 6.508255 | 7.617003 | 8.698512 | 9.825474 | 11.08469 | 12.75549 |
| **112** | 5.362597 | 6.733946 | 7.881142 | 9.000156 | 10.1662 | 11.46908 | 13.19782 |
| **113** | 5.539243 | 6.955765 | 8.14075 | 9.296625 | 10.50108 | 11.84688 | 13.63256 |
| **114** | 5.713067 | 7.17404 | 8.396211 | 9.588357 | 10.83061 | 12.21864 | 14.06036 |
| **115** | 5.884332 | 7.389102 | 8.647911 | 9.875796 | 11.15528 | 12.58493 | 14.48186 |
| **116** | 6.053409 | 7.601416 | 8.896394 | 10.15956 | 11.47581 | 12.94653 | 14.89797 |
| **117** | 6.220798 | 7.811611 | 9.142398 | 10.44049 | 11.79314 | 13.30453 | 15.30993 |
| **118** | 6.387013 | 8.020331 | 9.386676 | 10.71945 | 12.10825 | 13.66002 | 15.719 |
| **119** | 6.552563 | 8.228217 | 9.629977 | 10.9973 | 12.42209 | 14.01408 | 16.12643 |
| **120** | 6.717932 | 8.435875 | 9.873012 | 11.27484 | 12.73559 | 14.36776 | 16.53342 |
| **121** | 6.883549 | 8.643843 | 10.11641 | 11.5528 | 13.04956 | 14.72197 | 16.94102 |
| **122** | 7.049834 | 8.852651 | 10.36079 | 11.83188 | 13.36479 | 15.07761 | 17.35026 |
| **123** | 7.21721 | 9.06283 | 10.60678 | 12.11279 | 13.6821 | 15.43558 | 17.76219 |
| **124** | 7.386126 | 9.274942 | 10.85502 | 12.39629 | 14.00232 | 15.79684 | 18.1779 |
| **125** | 7.557108 | 9.489649 | 11.10631 | 12.68325 | 14.32647 | 16.16252 | 18.59871 |
| **126** | 7.730698 | 9.70763 | 11.36142 | 12.97459 | 14.65555 | 16.53378 | 19.02593 |
| **127** | 7.907437 | 9.929565 | 11.62117 | 13.27121 | 14.9906 | 16.91178 | 19.4609 |
| **128** | 8.087743 | 10.15598 | 11.88615 | 13.57382 | 15.33242 | 17.2974 | 19.90464 |
| **129** | 8.271474 | 10.3867 | 12.15618 | 13.88218 | 15.68073 | 17.69035 | 20.35682 |
| **130** | 8.458328 | 10.62133 | 12.43079 | 14.19579 | 16.03496 | 18.08998 | 20.81669 |
| **131** | 8.648002 | 10.85951 | 12.70954 | 14.51412 | 16.39454 | 18.49564 | 21.28349 |
| **132** | 8.840177 | 11.10083 | 12.99197 | 14.83665 | 16.75886 | 18.90665 | 21.75645 |
| **133** | 9.034413 | 11.34474 | 13.27743 | 15.16264 | 17.12708 | 19.32206 | 22.23448 |
| **134** | 9.230217 | 11.59061 | 13.56519 | 15.49126 | 17.49828 | 19.74083 | 22.71638 |
| **135** | 9.427097 | 11.83784 | 13.85454 | 15.82169 | 17.87152 | 20.1619 | 23.20091 |
| **136** | 9.624574 | 12.08582 | 14.14476 | 16.15312 | 18.24589 | 20.58425 | 23.68692 |
| **137** | 9.822342 | 12.33416 | 14.43541 | 16.48504 | 18.62081 | 21.00722 | 24.17365 |
| **138** | 10.02021 | 12.58263 | 14.72621 | 16.81713 | 18.99593 | 21.43041 | 24.66063 |
| **139** | 10.21801 | 12.831 | 15.0169 | 17.14909 | 19.37089 | 21.85343 | 25.14741 |
| **140** | 10.41554 | 13.07906 | 15.30721 | 17.48062 | 19.74537 | 22.27591 | 25.63357 |
| **141** | 10.61297 | 13.32698 | 15.59736 | 17.81197 | 20.11966 | 22.69816 | 26.11946 |
| **142** | 10.81077 | 13.57535 | 15.88805 | 18.14393 | 20.49462 | 23.12118 | 26.60625 |
| **143** | 11.0094 | 13.82478 | 16.17997 | 18.4773 | 20.87119 | 23.546 | 27.0951 |
| **144** | 11.20936 | 14.07588 | 16.47384 | 18.8129 | 21.25027 | 23.97367 | 27.58723 |
| **145** | 11.41153 | 14.32974 | 16.77095 | 19.1522 | 21.63352 | 24.40603 | 28.08477 |
| **146** | 11.61733 | 14.58817 | 17.07341 | 19.4976 | 22.02367 | 24.84619 | 28.59127 |
| **147** | 11.82827 | 14.85305 | 17.38342 | 19.85162 | 22.42356 | 25.29733 | 29.1104 |
| **148** | 12.04582 | 15.12624 | 17.70315 | 20.21675 | 22.83599 | 25.76262 | 29.64582 |
| **149** | 12.2714 | 15.4095 | 18.03467 | 20.59534 | 23.26363 | 26.24506 | 30.20099 |
| **150** | 12.50621 | 15.70436 | 18.37976 | 20.98943 | 23.70878 | 26.74726 | 30.77889 |
| **151** | 12.75144 | 16.0123 | 18.74016 | 21.40101 | 24.17368 | 27.27174 | 31.38242 |
| **152** | 13.00828 | 16.33482 | 19.11762 | 21.83206 | 24.66058 | 27.82104 | 32.01451 |
| **153** | 13.27772 | 16.67316 | 19.5136 | 22.28427 | 25.17137 | 28.39729 | 32.67763 |
| **154** | 13.56013 | 17.02779 | 19.92865 | 22.75824 | 25.70675 | 29.00129 | 33.37266 |
| **155** | 13.85573 | 17.39899 | 20.36308 | 23.25436 | 26.26715 | 29.6335 | 34.10017 |
| **156** | 14.16476 | 17.78705 | 20.81725 | 23.77301 | 26.853 | 30.29443 | 34.86073 |
| **157** | 14.48731 | 18.19207 | 21.29128 | 24.31435 | 27.46447 | 30.98427 | 35.65454 |
| **158** | 14.82288 | 18.61346 | 21.78445 | 24.87754 | 28.10062 | 31.70195 | 36.4804 |
| **159** | 15.17073 | 19.05026 | 22.29567 | 25.46135 | 28.76007 | 32.44592 | 37.3365 |
| **160** | 15.53014 | 19.50158 | 22.82388 | 26.06455 | 29.44142 | 33.21459 | 38.22104 |
| **161** | 15.90035 | 19.96647 | 23.36796 | 26.68589 | 30.14325 | 34.00637 | 39.13216 |
| **162** | 16.28041 | 20.44371 | 23.9265 | 27.32374 | 30.86375 | 34.8192 | 40.06751 |
| **163** | 16.66924 | 20.93198 | 24.49795 | 27.97633 | 31.60088 | 35.6508 | 41.02446 |
| **164** | 17.06578 | 21.42993 | 25.08074 | 28.64186 | 32.35264 | 36.4989 | 42.00039 |
| **165** | 17.46898 | 21.93623 | 25.67329 | 29.31854 | 33.11699 | 37.36121 | 42.99268 |
| **166** | 17.87778 | 22.44958 | 26.27409 | 30.00465 | 33.89199 | 38.23553 | 43.99879 |
| **167** | 18.2912 | 22.96871 | 26.88166 | 30.69849 | 34.67572 | 39.11971 | 45.01624 |
| **168** | 18.70821 | 23.49236 | 27.49453 | 31.39837 | 35.46628 | 40.01158 | 46.04255 |
| **169** | 19.12783 | 24.01929 | 28.11122 | 32.10263 | 36.26178 | 40.90903 | 47.07527 |
| **170** | 19.54928 | 24.54852 | 28.73061 | 32.80996 | 37.06075 | 41.81039 | 48.11249 |
| **171** | 19.97206 | 25.07942 | 29.35195 | 33.51952 | 37.86224 | 42.71461 | 49.153 |
| **172** | 20.39569 | 25.61138 | 29.97454 | 34.23051 | 38.66535 | 43.62064 | 50.1956 |
| **173** | 20.81969 | 26.1438 | 30.59767 | 34.94212 | 39.46915 | 44.52745 | 51.23909 |
| **174** | 21.24374 | 26.67629 | 31.22087 | 35.6538 | 40.27304 | 45.43437 | 52.28271 |
| **175** | 21.66782 | 27.20882 | 31.84412 | 36.36554 | 41.07699 | 46.34135 | 53.3264 |
| **176** | 22.09193 | 27.74139 | 32.46741 | 37.07734 | 41.88101 | 47.24841 | 54.37018 |
| **177** | 22.51609 | 28.27402 | 33.09078 | 37.78922 | 42.68511 | 48.15557 | 55.41408 |
| **178** | 22.94031 | 28.80671 | 33.71423 | 38.50119 | 43.48932 | 49.06284 | 56.45811 |
| **179** | 23.36456 | 29.33945 | 34.33773 | 39.21321 | 44.2936 | 49.9702 | 57.50222 |
| **180** | 23.78883 | 29.87222 | 34.96126 | 39.92528 | 45.09792 | 50.87759 | 58.54639 |

1. Handgrip strength for age for girls

| Age | **2^nd^** | **9^th^** | **25^th^** | **50^th^** | **75^th^** | **91^st^** | **98^th^** |
| --- | --- | --- | --- | --- | --- | --- | --- |
| 5 | 4.299539 | 5.273447 | 6.265149 | 7.357302 | 8.549428 | 9.839886 | 11.36655 |
| 6 | 5.308145 | 6.510517 | 7.734857 | 9.083213 | 10.55499 | 12.14817 | 14.03296 |
| 7 | 6.330181 | 7.764059 | 9.224134 | 10.8321 | 12.58726 | 14.4872 | 16.73489 |
| 8 | 7.388882 | 9.062571 | 10.76684 | 12.64374 | 14.69244 | 16.91013 | 19.53374 |
| 9 | 8.493852 | 10.41783 | 12.37697 | 14.53454 | 16.88962 | 19.43895 | 22.45491 |
| 10 | 9.636778 | 11.81965 | 14.0424 | 16.4903 | 19.16227 | 22.05464 | 25.47642 |
| 11 | 10.79286 | 13.2376 | 15.727 | 18.46856 | 21.46108 | 24.70044 | 28.53271 |
| 12 | 11.94479 | 14.65046 | 17.40556 | 20.43974 | 23.75165 | 27.33675 | 31.57805 |
| 13 | 13.11024 | 16.0799 | 19.10382 | 22.43404 | 26.06909 | 30.00399 | 34.65911 |
| 14 | 14.30556 | 17.54598 | 20.8456 | 24.47945 | 28.44593 | 32.73959 | 37.81914 |
| 15 | 15.52827 | 19.04565 | 22.6273 | 26.57173 | 30.87723 | 35.53787 | 41.05157 |
| 16 | 16.76542 | 20.56303 | 24.43003 | 28.68872 | 33.33724 | 38.36919 | 44.32217 |

1. Handgrip strength for age for boys

| **Age** | **2^nd^** | **9^th^** | **25^th^** | **50^th^** | **75^th^** | **91^st^** | **98^th^** |
| --- | --- | --- | --- | --- | --- | --- | --- |
| **5** | 4.390597 | 5.944898 | 7.229256 | 8.450966 | 9.668809 | 10.94123 | 12.47478 |
| **6** | 5.361821 | 7.259942 | 8.828407 | 10.32037 | 11.8076 | 13.36149 | 15.23427 |
| **7** | 6.333638 | 8.575789 | 10.42853 | 12.19091 | 13.9477 | 15.78323 | 17.99545 |
| **8** | 7.215715 | 9.770127 | 11.8809 | 13.88872 | 15.89018 | 17.98133 | 20.50165 |
| **9** | 8.150031 | 11.0352 | 13.41928 | 15.68708 | 17.94769 | 20.30962 | 23.15627 |
| **10** | 9.150015 | 12.38918 | 15.06579 | 17.61183 | 20.14982 | 22.80155 | 25.99748 |
| **11** | 10.20114 | 13.81241 | 16.7965 | 19.63503 | 22.46457 | 25.42092 | 28.98399 |
| **12** | 11.48091 | 15.54523 | 18.90368 | 22.09831 | 25.28283 | 28.61007 | 32.62013 |
| **13** | 13.2699 | 17.96753 | 21.8493 | 25.54173 | 29.22247 | 33.06816 | 37.70309 |
| **14** | 15.40676 | 20.86086 | 25.36772 | 29.65474 | 33.9282 | 38.39317 | 43.77446 |
| **15** | 17.66871 | 23.92356 | 29.09209 | 34.00852 | 38.90938 | 44.02988 | 50.20123 |
| **16** | 19.92528 | 26.97896 | 32.80759 | 38.35192 | 43.8787 | 49.65316 | 56.61269 |


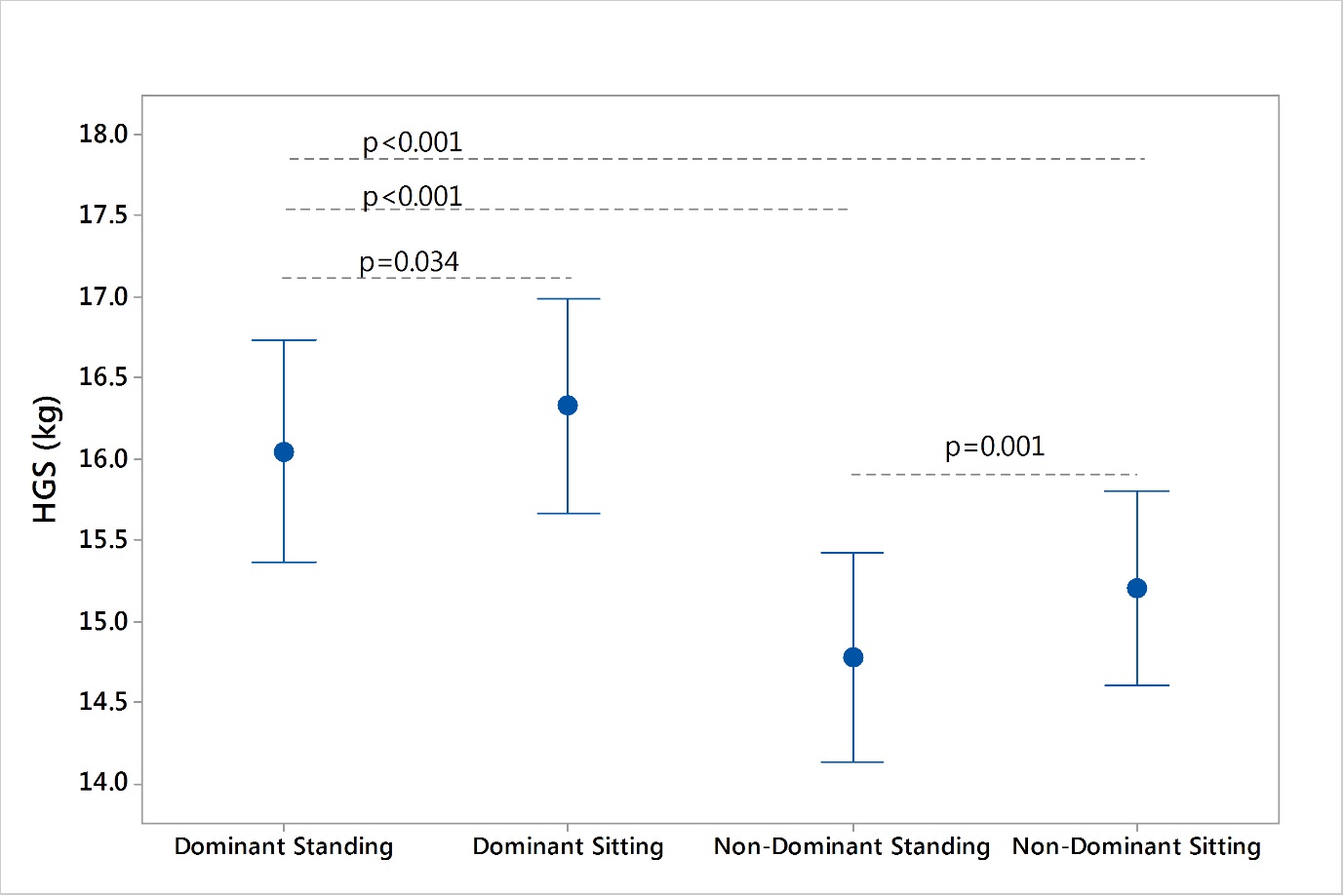


**Supplementary Figure 1:** Handgrip strength (kg) compared between dominant and non-dominant hands while sitting and while standing.


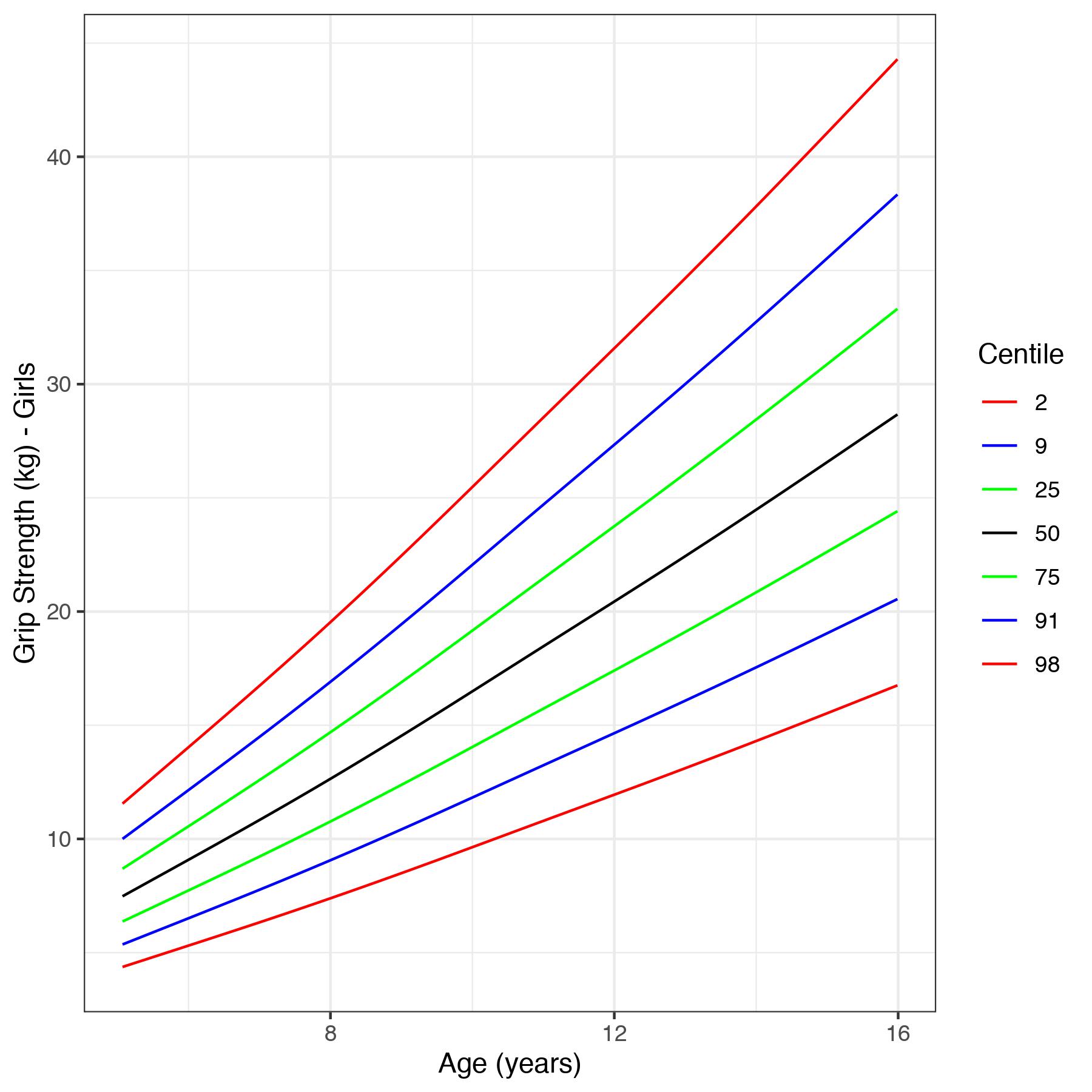


**Supplementary Figure 2**: HGS (kg) centile chart adjusted for age (years) in girls.


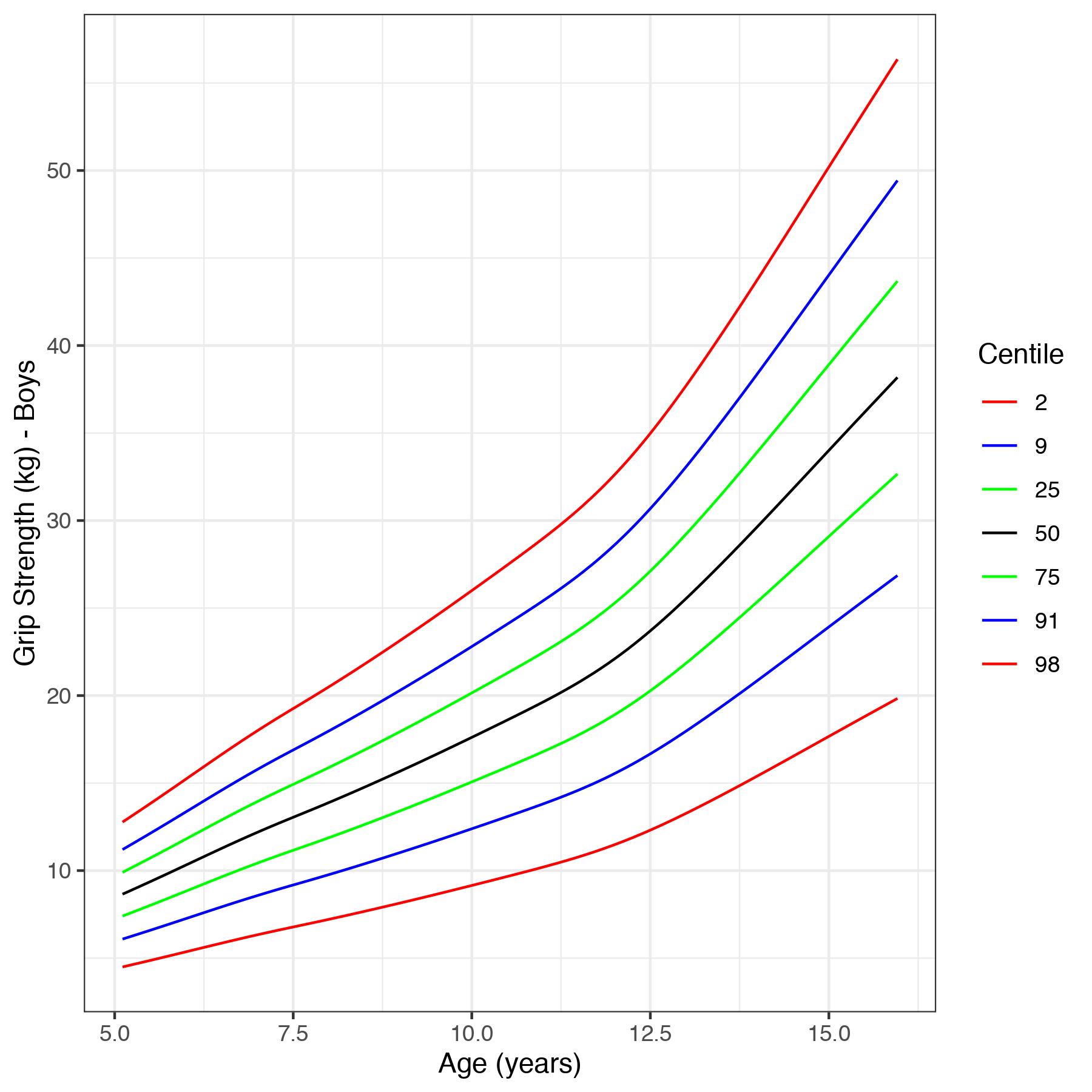


**Supplementary Figure 3:** HGS (kg) centile chart adjusted for age (years) in boys.


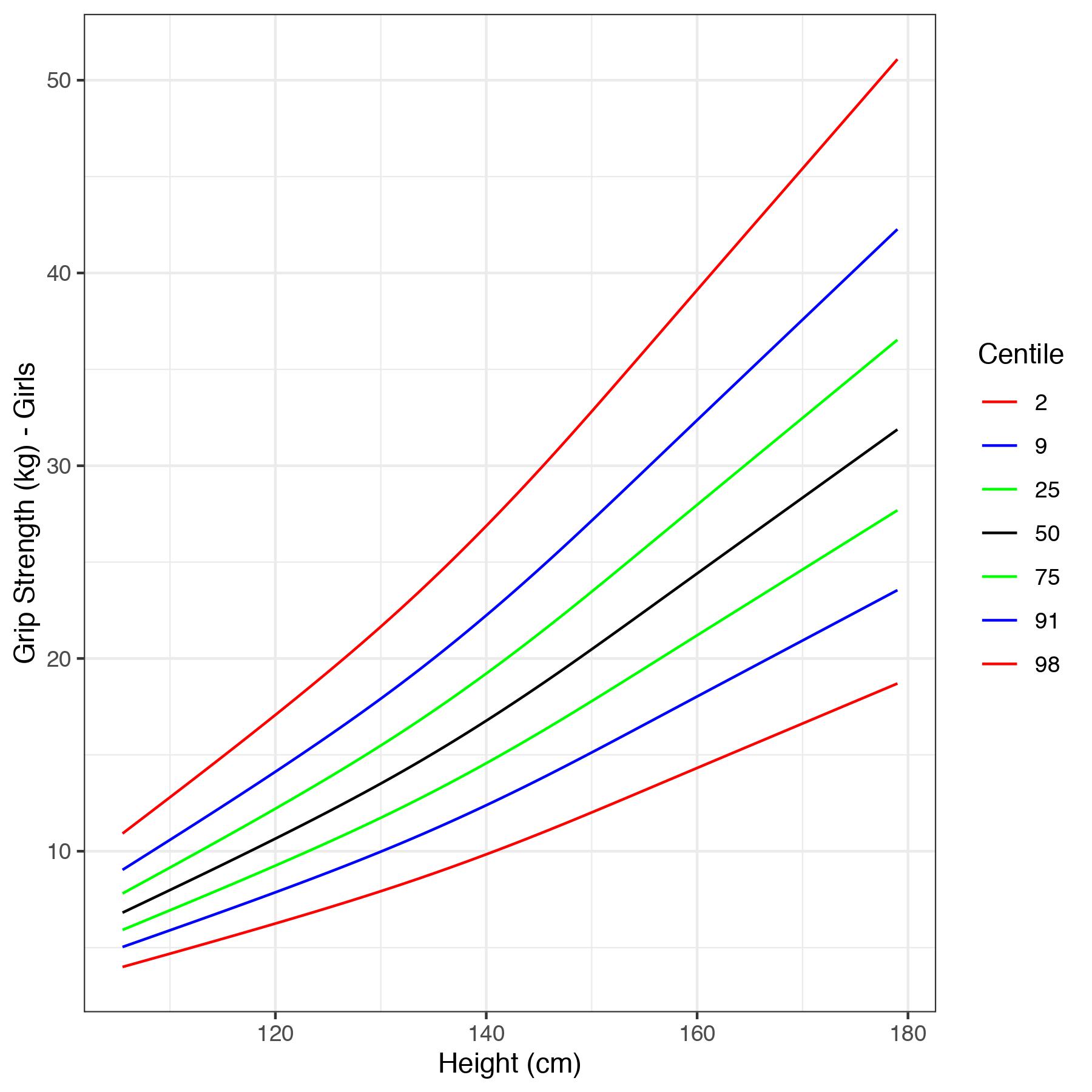


**Supplementary Figure 4:** HGS (kg) centile chart adjusted for height (cm) in girls.


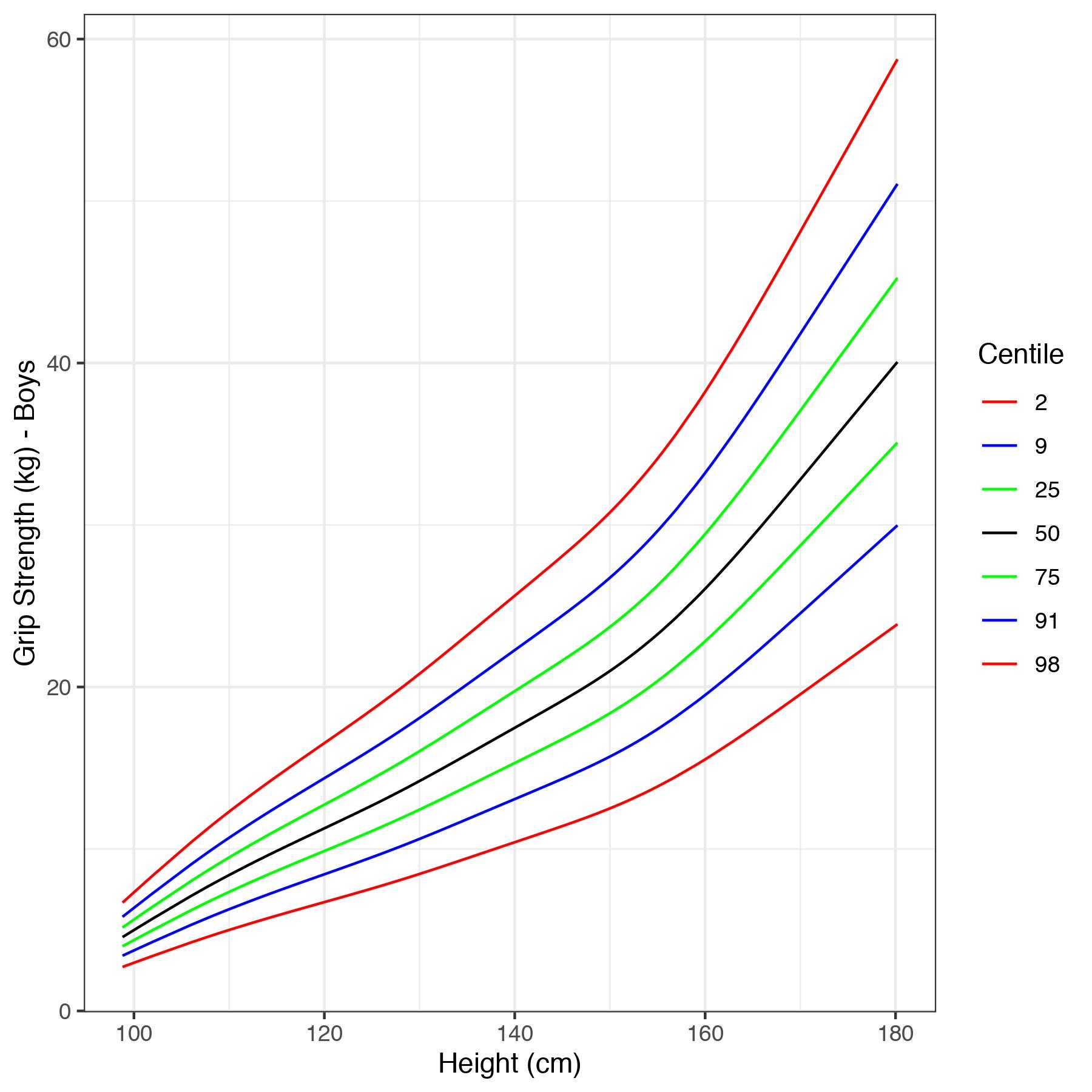


**Supplementary Figure 5:** HGS (kg) centile chart adjusted for height (cm) in boys.


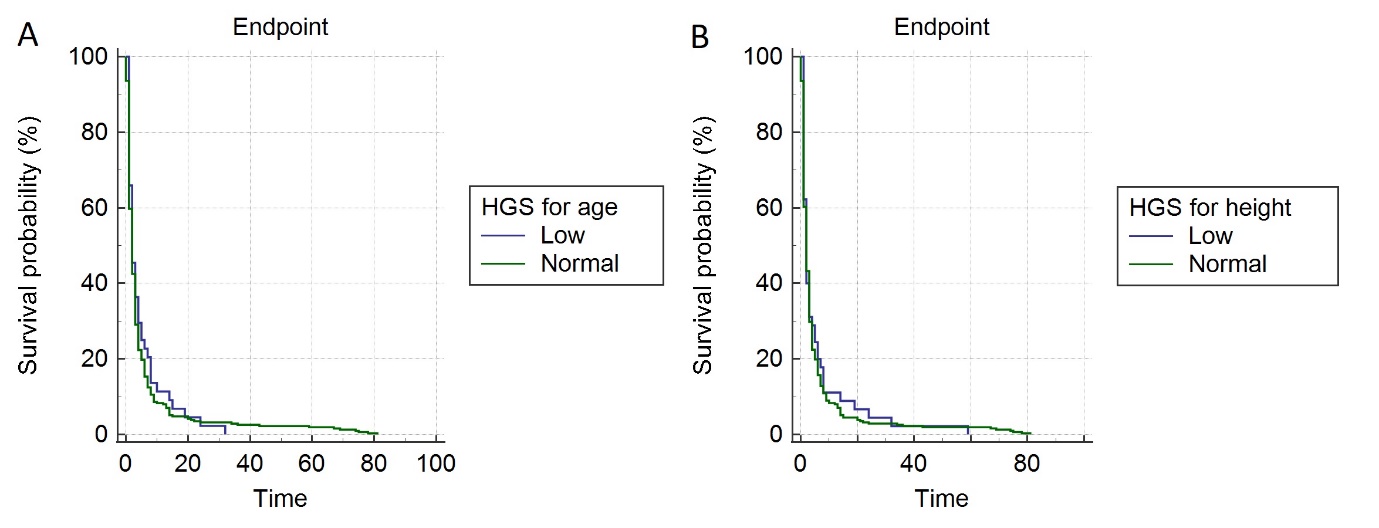


**Supplementary Figure 6**: Kaplan Meier Curve for handgrip strength adjusted for age (A) and height (B) and length of hospital stay in sick children.


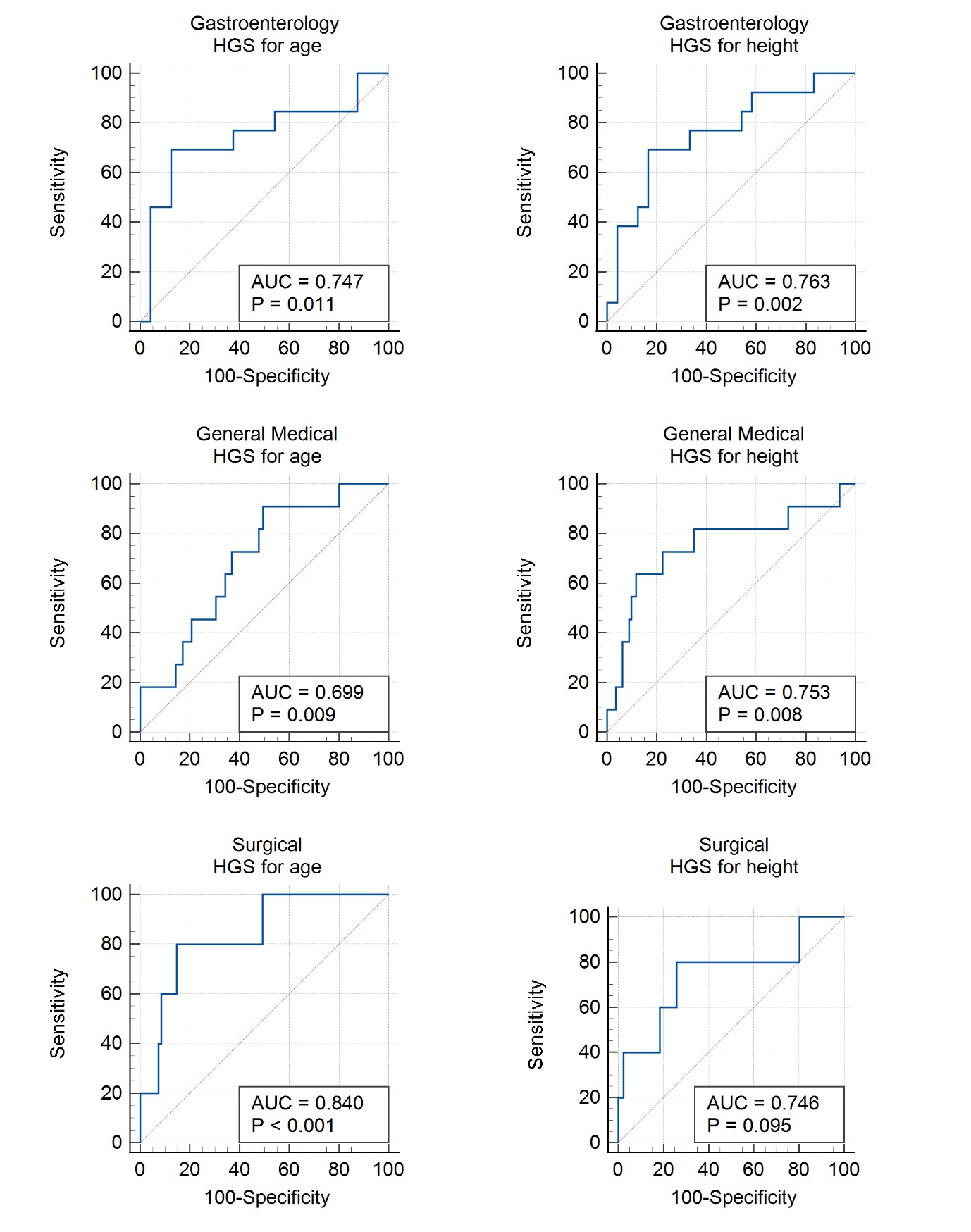


**Supplementary Figure 7:** ROC curve for handgrip strength adjusted for age and height as a tool to detect high malnutrition risk in sick children within gastroenterology, general medical, and surgical specialties.
